# Canonical correlations reveal co-variability between spike trains and local field potentials in area MT)

**DOI:** 10.1186/1471-2202-16-S1-P194

**Published:** 2015-12-18

**Authors:** Jacob Yates, Evan Archer, Alexander C Huk, Il Memming Park

**Affiliations:** 1 Center for Perceptual Systems, The University of Texas at Austin, Austin, TX 78712, USA; 2 Department of Statistics and Grossman Center for the Statistics of Mind, Columbia University, New York, NY 10027, USA; 3 Department of Neurobiology and Behavior, Stony Brook University, Stony Brook, NY 11794, USA

## 

Patterns of neuronal correlations can provide important clues about the structure of the underlying network and how it processes information. Several recent studies have found that neural population activity across a region can be explained in large part by a shared, low-dimensional signal [1-5]. Population-wide correlation is likely to influence the local field potential (LFP) - an epiphenomenon that reflects low-frequency, concerted neural activity from anatomically connected circuits. Here, we show that LFP and spike trains recorded simultaneously from the middle temporal (MT) area of the awake macaque indeed share population-wide correlation. We apply canonical correlation analysis (CCA) to 16 channels of LFP and 16 spike sorted neurons (from 12 channels) acquired at 50 ms temporal resolution during inter-trial intervals (when the monkey was free to make eye movements), as well as during performance of a perceptual decision-making task (when the monkey maintained fixation and discriminated the direction of visual motion). CCA finds instantaneous linear projections of the LFP that maximize the correlation to corresponding projections of the population spike trains.

Previous studies have suggested using population spike rate as a proxy for the local network state [3,5]. Applied to our dataset, we obtain a correlation coefficient of -12% between population spike rate and the mean LFP during inter-trial interval segments. In contrast, we obtain pairs of canonical variables with corresponding canonical correlations 29%, 26%, and 21%. We then applied the extracted projections to the task-relevant motion stimulus integration window. We find that the correlation of the projections is maintained for the 1st (31%) and 3rd (18%) components, but drops significantly for the 2nd component (7%)-- indicating a task-specific decoupling of LFP and spikes in a subspace uncovered by CCA. Upon further analysis, each CCA projection showed a distinct stimulus encoding pattern in spike rate and LFP. We hypothesize that CCA projections reveal functional, virtual units of information processing.

The LFP is an important source of information when neural activity is correlated. It can indicate the strength of correlations, and the common input giving rise to such correlations. Additionally, the LFP provides increased statistical power to analyses, especially in areas where large-scale recording is anatomically difficult. CCA is a simple technique that can reveal low-dimensional structure in the data, uncovering components which maximize covariability between LFP and spike trains within MT.
